# Efficacy and Safety of Intravenous Diltiazem Versus Metoprolol in the Management of Atrial Fibrillation with Rapid Ventricular Response in the Emergency Department: A Comprehensive Umbrella Review of Systematic Reviews and Meta-analyses

**DOI:** 10.19102/icrm.2024.15095

**Published:** 2024-09-15

**Authors:** Fnu Jaya, Maria Afzal, Fnu Anusha, Muskan Kumari, Ajay Kumar, Saqib Saleem, Aman Kumar, Vishal Bhatia, Rabia Islam, Manoj Kumar, Rameet Kumar, Hamza Islam, Muhammad Ali Muzammil, Satesh Kumar, Mahima Khatri

**Affiliations:** 1 Department of Medicine, Ziauddin University Hospital, Karachi, Pakistan; 2 Department of Medicine, Bahria University of Medical and Dental College, Karachi, Pakistan; 3 Department of Medicine, Ghulam Muhammad Mahar Medical College, Sukkur, Pakistan; 4 Department of Medicine, Chandka Medical College SMBBMU Larkana, Larkana, Pakistan; 5 Department of Medicine, Khairpur Medical College, Khaipur, Pakistan; 6 Department of Medicine, Punjab Medical College, Faisalabad, Pakistan; 7 Department of Medicine, Dow University of Health Sciences, Karachi, Pakistan; 8 Department of Medicine, Shaheed Mohtarma Benazir Bhutto Medical College, Karachi, Pakistan

**Keywords:** Acute rate control, atrial fibrillation, comparison, intravenous diltiazem, metoprolol, rapid ventricular response

## Abstract

Atrial fibrillation (AF) is the most common cardiac arrhythmia in the United States, affecting 2.7–6.1 million people. AF can cause symptoms, but when it triggers a rapid ventricular response (RVR), most patients suffer from decompensation. Therefore, we performed an umbrella review of systematic reviews and meta-analyses comparing intravenous (IV) metoprolol and diltiazem to identify discrepancies, fill in knowledge gaps, and develop standardized decision-making guidelines for physicians to manage AF with RVR. A comprehensive search was conducted in PubMed, the Cochrane Library, and Scopus to identify studies for this umbrella review. The overall certainty of the evidence was assessed using the Grading of Recommendations Assessment, Development, and Evaluation method, while the quality of the included reviews was evaluated using AMSTAR 2, the Cochrane Collaboration tool, and the Newcastle–Ottawa scale. This study comprehensively analyzed four meta-analyses covering 11 randomized controlled trials and 19 observational studies. The analysis showed that IV diltiazem treatment was significantly more successful in rate control for AF with rapid ventricular response (RVR) than IV metoprolol (risk ratio [RR], 1.30; 95% confidence interval [CI], 1.09–1.56; *I*^2^ = 0%; *P* = .003). IV diltiazem also led to a significantly greater reduction in ventricular rate (mean difference, −14.55; 95% CI, −16.93 to −12.16; *I*^2^ = 72%; *P* < .00001), particularly at 10 min. The analysis also revealed a significantly increased risk of hypotension associated with treatment with IV diltiazem (RR, 1.43; 95% CI, 1.14–1.79; *I*^2^ = 0%; *P* = .002). In conclusion, IV diltiazem therapy achieved better rate control and ventricular rate decrease than metoprolol therapy in AF with RVR. Future clinical trials should compare calcium channel blockers and β-blockers for heart rate control efficacy and safety, considering adverse events.

## Introduction

Atrial fibrillation (AF) is the most common cardiac arrhythmia in the United States, affecting 2.7–6.1 million people.^1^ In 2017, AF was identified as the major cause of death among 26,077 individuals. In 2014, AF caused >500,000 emergency department (ED) visits, a 30.7% increase from 2007.^2^ This spike in incidence emphasizes the significance of treating AF, the most commonly treated arrhythmia in the ED. AF can cause different symptoms; however, most individuals decompensate when it causes a rapid ventricular response (RVR). Symptomatic AF with RVR is caused by reduced cardiac output due to a decrease in ventricular diastolic filling time and the absence of the atrial kick, both of which are required for adequate ventricular filling.^3^

There are two basic ED treatments for AF with RVR. These methods regulate the heart’s rhythm via electrical or chemical cardioversion, which requires anti-arrhythmics. Alternatively, rate control can be achieved via medications that influence the function of the atrioventricular (AV) node.^4^ Hemodynamically stable patients without urgent electrical cardioversion can be managed with pharmacological rate or rhythm management. Regardless of modality, a prolonged continuous accelerated ventricular rate might lead to left ventricular dysfunction due to tachycardia, emphasizing the urgency of this disease.^5^ The latest RVR recommendations for treating AF focus on rate-regulating medications to lower asymptomatic individuals’ heart rates (HRs) to <110 bpm. The European Society of Cardiology (ESC), the American Heart Association (AHA), the American College of Cardiology (ACC), and the Heart Rhythm Society (HRS) recommend starting rate control with non-dihydropyridine calcium channel blockers or intravenous (IV) β-blockers.^6^

Hospital preferences, patient comorbidities, and prior use of home medications may influence medication selection. Acutely decompensated systolic heart failure (HF) patients should avoid non-dihydropyridine calcium channel blockers.^7^ There is a lack of clarity regarding the usefulness and safety of β-blockers and calcium channel blockers for patients with an RVR who do not have specific reasons to avoid them.^8^ When treating AF with RVR, many ED doctors use diltiazem to regulate the rate. Diltiazem treats AF with RVR by inhibiting AV node conduction and prolonging its refractory time.^9^ Diltiazem bolus has been shown to control ventricular rate better than metoprolol or digoxin within 30 min. Nonetheless, there is a scarcity of data that directly compare the use of IV diltiazem with metoprolol for the urgent treatment of RVR in patients with AF and HF.^9,10^ Therefore, we performed an umbrella review of systematic reviews and meta-analyses comparing IV metoprolol and diltiazem to identify discrepancies, fill knowledge gaps, and develop standardized decision-making guidelines for physicians to manage AF with RVR.

## Methodology

This umbrella review, adhering to the guidelines outlined in the Preferred Reporting Items for Systematic Reviews and Meta-Analysis (PRISMA) and the Cochrane Collaboration Handbook,^11,12^ systematically examined existing systematic reviews and meta-analyses.

### Search strategy

The search strategy was meticulously implemented across reputable databases, including PubMed, Cochrane Library, and Scopus, to guarantee a thorough exploration of the literature. The search incorporated all the relevant keywords and Medical Subject Headings to ensure inclusivity. The detailed search strategy, including the specific combinations of keywords and operators, is briefly summarized in **Supplementary Table S1**. To mitigate potential selection bias, the search process was independently conducted by two researchers, with any disagreements resolved through consensus. In cases where discrepancies persisted, a third researcher was consulted for resolution.

### Study inclusion and exclusion criteria

#### Inclusion criteria

Studies that specifically target adult participants aged ≥18 years who have been diagnosed with AF were included. Reviews exploring IV diltiazem as a therapeutic approach for AF were incorporated. Additionally, meta-analyses and systematic reviews directly comparing the efficacy and safety of IV diltiazem with IV metoprolol for treating AF with RVR were considered. Studies that provided insights into relevant clinical outcomes, such as rate control success at different time intervals and adverse events, were also considered.

#### Exclusion criteria

Primary studies, conference abstracts, letters, editorials, and reviews failing to meet the criteria for systematic reviews and meta-analyses were excluded from consideration. Reviews focusing on pediatric populations (participants < 18 years old) or using animal models were also excluded. Studies examining treatments unrelated to IV diltiazem or IV metoprolol for AF with RVR were not considered. Furthermore, reviews that did not directly compare IV diltiazem with IV metoprolol in the context of AF were excluded. This umbrella review did not include studies lacking relevant clinical outcomes or reporting incomplete data.

### Data extraction and definitions

In the process of data extraction, pertinent information was collected, encompassing publication details (title, authors, publication year, and source), study attributes (design, number of primary studies included), participant features (age range, gender distribution), and particulars concerning the doses of IV diltiazem and IV metoprolol. Additionally, outcome measures were extracted, composing rate control success at various time intervals (5, 10, 30, and 60 min); a decrease in ventricular rate at distinct time points (5, 10, 15, 30, and 60 min); and occurrences of hypotension, bradycardia, and other adverse events (nausea, headache, swelling, and rash). Effect measures employed in the comparisons, such as risk ratios (RRs) and mean differences (MDs), were recorded, along with details of the quality assessment methodology and findings.

The outcomes were stratified into two categories: efficacy outcomes and safety outcomes. Efficacy outcomes included rate control success at different time intervals (5, 10, 30, and 60 min) and a decreased ventricular rate at distinct time points (5, 10, 15, 30, and 60 min). Safety outcomes included hypotension, bradycardia, and other adverse events (nausea, headache, swelling, and rash). These categories were further divided into primary efficacy outcomes (rate control success at different time intervals), secondary efficacy outcomes (decrease in ventricular rate at different time intervals), primary safety outcomes (hypotension), and secondary safety outcomes (bradycardia and other adverse events).

Rate control success was defined as achieving an HR of <100 bpm or a decrease of ≥20% from the baseline HR in patients hospitalized with AF with rapid ventricular response (RVR). Hypotension was characterized by a systolic blood pressure of <90 mmHg, while bradycardia was delineated as an HR of <60 bpm.

### Assessment of risk of bias

Two investigators autonomously evaluated the methodological quality of the incorporated reviews and meta-analyses using the AMSTAR 2 tool.^13^ Furthermore, the risk of bias in the randomized controlled trials (RCTs) included in individual meta-analyses was appraised using the Cochrane Collaboration risk of bias tool.^14^ The quality of observational studies was assessed using the Newcastle–Ottawa scale, considering domains such as study design, participant selection, blinding, outcome reporting, and other pertinent parameters.^15^

The certainty of evidence and the strength of recommendations derived from meta-analyses were evaluated using the Grading of Recommendations Assessment, Development, and Evaluation (GRADE) method.^16^ Two researchers independently conducted the GRADE assessment with discussions and agreements to resolve discrepancies.

### Statistical analysis

All statistical analyses were conducted using Comprehensive Meta-Analysis Software version 4 and Review Manager version 5.4.1, which are both tools developed by The Nordic Cochrane Centre in collaboration with the Cochrane Collaboration in Denmark in 2014. To assess categorical outcomes, we reassessed effect sizes as RRs with corresponding 95% confidence intervals (CIs) using the DerSimonian and Laird random-effects model. MDs were computed for continuous data. Subgroup analysis was employed for the two efficacy outcomes, using different time intervals as subgroups to understand the effects of the two drugs at various time points. Statistical significance in two-sided tests was considered at *P* < .05. The *I*^2^ statistic was applied to evaluate the heterogeneity between study associations.^17^ Sensitivity analyses were undertaken to appraise the robustness of summary estimates and identify any individual study significantly contributing to heterogeneity if the heterogeneity was >75%. Egger’s regression asymmetry test was employed to scrutinize the evidence of small-study effects,^18^ with *P* < .05 indicating such effects. “*P*-hacking”^19^ and an assessment of publication bias were conducted through funnel plots.

Regarding ethical considerations and conflicts of interest, as this umbrella review solely relies on previously published systematic reviews and meta-analyses, it did not involve collecting or analyzing primary data from human participants. Therefore, ethical review board approval and patient consent do not apply to this study.

## Results

### Study selection

Initially, 20 systematic reviews and meta-analyses were identified, and duplicate entries were removed. After thoroughly examining the complete texts, we ultimately incorporated four systematic reviews and meta-analyses.^20–23^ These selected studies collectively encompassed data from 11 RCTs and 19 observational studies. **Table 1** briefly summarizes the essential characteristics of the meta-analyses integrated into this review.

### Risk of bias of included studies

**Supplementary Figure S1:**
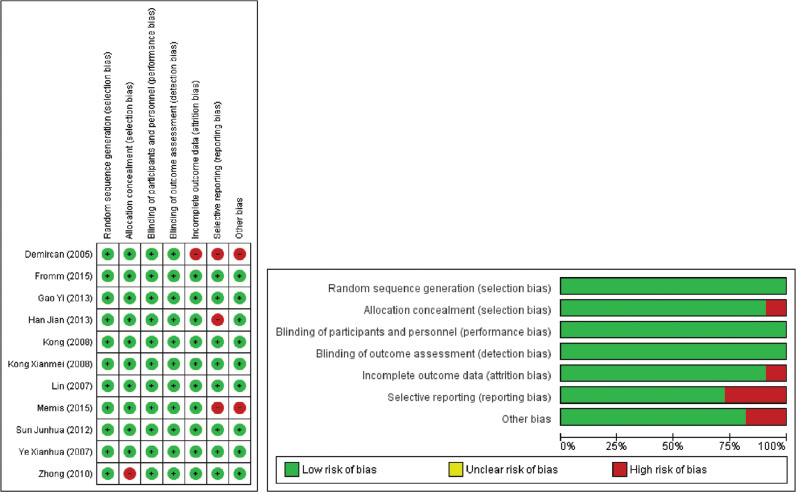
Cochrane risk-of-bias assessment for individual randomized controlled trials.

The methodological quality ratings for the four systematic reviews and meta-analyses, assessed through the AMSTAR 2 tool, are detailed in **Supplementary Table S2**. All four studies received a moderate quality grade. The GRADE assessment, as outlined in **Supplementary Table S3**, indicated a varied level of certainty in the reviews used for our study, ranging from moderate to high. Individual RCTs underwent quality assessment using the Cochrane risk of bias tool, revealing trials with a moderate to low risk of bias, as depicted in **Supplementary Figure S1**. Additionally, the quality evaluation of observational studies was conducted using the Newcastle–Ottawa scale, revealing a spectrum of quality among the studies included in the analysis, spanning from fair to good, as elucidated in **Supplementary Table S4**.

### Synthesis of results

#### Primary efficacy outcome

**Figure 1:**
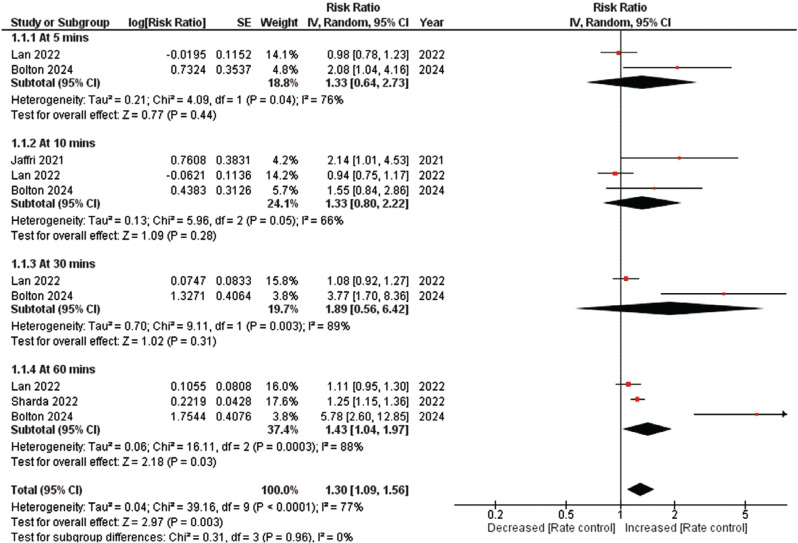
Rate control success. The figure illustrates the results of a comprehensive analysis comparing the rate control success at different time intervals between intravenous diltiazem and intravenous metoprolol in patients with atrial fibrillation and rapid ventricular response. The analysis indicates significantly greater rate control success with intravenous diltiazem, which was most evident at 30 min. *Abbreviations:* CI, confidence interval; IV, inverse variance; SE, standard error.

The primary efficacy outcome under investigation focused on the success of rate control at different time intervals. The comprehensive analysis revealed that IV diltiazem treatment was linked to a significantly greater occurrence of rate control success in patients with AF with RVR compared to IV metoprolol (RR, 1.30; 95% CI, 1.09–1.56; *I*^2^ = 0%; *P* = .003), as illustrated in **Figure 1**. Subsequently, a subgroup analysis was performed to better understand the efficacy of the two drugs at varying time intervals. The findings indicated that IV diltiazem was associated with the highest likelihood of rate control success at 30 min (RR, 1.89; 95% CI, 0.56–6.42; *P* = .31), although this result was statistically non-significant. Meanwhile, the second-highest likelihood of rate control success was noted at 60 min (RR, 1.43; 95% CI, 1.04–1.97; *P* = .03). Finally, the risk of rate control success was comparable at 5 min (RR, 1.33; 95% CI, 0.64–2.73; *P* = .44) and 10 min (RR, 1.33; 95% CI, 0.80–2.22; *P* = .31), respectively, and was statistically non-significant.

#### Secondary efficacy outcome

**Figure 2:**
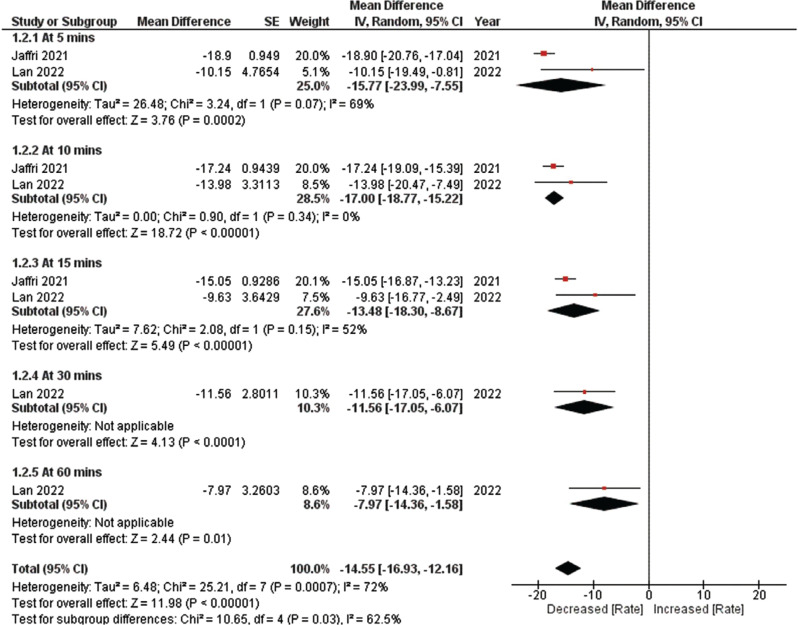
Ventricular rate reduction. This figure presents a comprehensive analysis comparing the reduction in ventricular rate at various time intervals between intravenous diltiazem and intravenous metoprolol in patients with acute atrial fibrillation. The analysis reveals a significantly greater reduction in ventricular rate with intravenous diltiazem, with the greatest reduction occurring at 10 min. *Abbreviations:* CI, confidence interval; IV, inverse variance; MD, mean difference; SE, standard error.

The secondary efficacy outcome focused on the reduction in ventricular rate at various time intervals. The comprehensive analysis indicated that IV diltiazem treatment was linked to a significantly greater reduction in ventricular rate compared to IV metoprolol (MD, −14.55; 95% CI, −16.93 to −12.16; *I*^2^ = 72%; *P* < .00001), as depicted in **Figure 2**. Moreover, a subgroup analysis conducted at different time intervals demonstrated that IV diltiazem treatment was significantly associated with the greatest reduction at 10 min (MD, −17.00; 95% CI, −18.77 to −15.22; *P* < .00001).

#### Primary safety outcome

**Figure 3:**
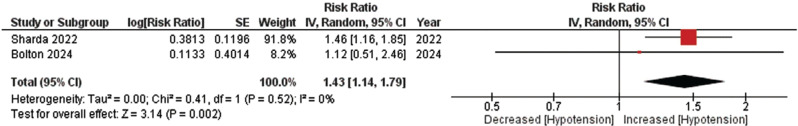
Hypotension. This figure illustrates the comprehensive analysis of hypotension incidence, comparing intravenous diltiazem and intravenous metoprolol in acute atrial fibrillation. The analysis reveals a significantly higher risk of hypotension associated with intravenous diltiazem treatment compared to intravenous metoprolol. *Abbreviations:* CI, confidence interval; IV, inverse variance; RR, risk ratio; SE, standard error.

The primary safety outcome, encompassing hypotension, was reported by two out of the four studies included in this review. The comprehensive analysis indicated that IV diltiazem treatment was linked to a significantly higher risk of hypotension compared to IV metoprolol (RR, 1.43; 95% CI, 1.14–1.79; *I*^2^ = 0%; *P* = .002), as illustrated in **Figure 3**.

#### Secondary safety outcomes

The secondary safety outcomes encompassed bradycardia and other adverse events, which were reported by one of the four studies. The study by Sharda and Bhatia^22^ provided data on bradycardia, revealing that IV diltiazem treatment was significantly associated with a higher risk of bradycardia compared to IV metoprolol (RR, 2.19; 95% CI, 1.22–3.94; *P* = .009). Similarly, data on other adverse events were reported by the study conducted by Lan et al.,^20^ indicating that IV diltiazem treatment was linked to a non-significantly reduced risk of other adverse events compared to IV metoprolol (RR, 0.80; 95% CI, 0.55–1.14; *P* = .09). Due to these outcomes being reported by only one meta-analysis, the results could not be pooled, and consequently, forest plots were not generated.

### P-hacking, publication bias, and small-study effect

Our study’s absence of *P*-hacking evidence indicates that the results were not manipulated to achieve a predetermined outcome. It is important to note that our assessment of publication bias was limited to two outcomes: rate control success at 10 and 60 min, respectively. This limitation arises because a minimum of three studies is required to conduct a comprehensive analysis using a funnel plot. A thorough assessment of publication bias for the remaining outcomes was not feasible, as each was reported by only one or two studies.

**Supplementary Figure S2:**
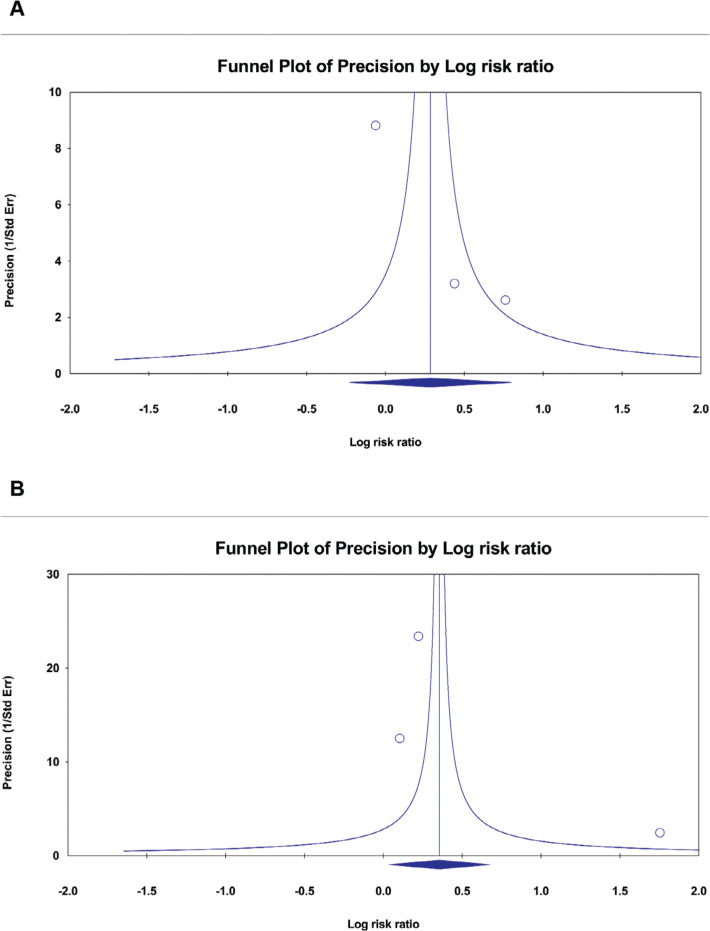
Funnel plots. **A:** Rate control success at 10 min. **B:** Rate control success at 60 min. The funnel plots showed no risk of publication bias. *Abbreviation:* Std Error, standard error.

In examining rate control success at 10 and 60 min, we conducted an in-depth analysis with an adequate number of studies to enable a funnel plot analysis. Our findings revealed a symmetrical distribution of data points in the funnel plot. The observed symmetry in the data suggest the absence of publication bias, as illustrated in **Supplementary Figure S2**. Additionally, we evaluated small-study effects using Egger’s regression asymmetry test. The results of our investigation indicated *P* values of .06 for rate control success at 10 min and .49 for rate control at 60 min, with both exceeding .05. This suggests a lack of substantial evidence for small-study effects.

## Discussion

AF is the most common abnormal heart rhythm that requires immediate attention in the ED, accounting for approximately one-third of hospital admissions related to cardiac arrhythmias.^24^ Historically, the ED has used two primary approaches to handle AF with the RVR: rhythm control, which involves techniques such as electrical cardioversion or anti-arrhythmic medications, and rate control using AV nodal-blocking agents.^24^ Both β-blockers and calcium channel blockers are commonly used to treat AF with RVR. However, it is still unclear which medication best benefits acute rate control.^25^ Metoprolol, a β-blocker, specifically blocks β1 receptors in the heart tissue, resulting in adverse inotropic and chronotropic effects similar to those of diltiazem. This inhibition leads to a decrease in the amount of blood pumped by the heart, which helps relieve symptoms of angina by reducing the amount of oxygen needed by the heart muscle.^26^ Diltiazem demonstrates higher initial effectiveness; however, this advantage disappears after 30 min. Research consistently shows a similar effectiveness in controlling HR between diltiazem and metoprolol within 30–60 min after taking the medication. Assessing long-term management is essential because there may be delays in giving oral treatment in the emergency room, which increases the likelihood of RVR recurring, depending on the duration of medication.^27^ Both drugs can be administered IV or orally, allowing for a smooth transition for patients after the desired drop in HR is reached. The onset of diltiazem is shorter, at 3 min, in contrast to metoprolol’s onset of 20 min following IV administration. The first delivery of diltiazem consists of an IV injection of 0.25 mg/kg, followed by a continuous infusion of 5–15 mg/h to reduce the ventricular rate.^28^ Continuous injection is restricted to a maximum duration of 24 h to avoid drug buildup. Unlike metoprolol, which cannot be supplied continuously, diltiazem is given via an IV push at a dose of 2.5–5 mg.^28^

Our umbrella review consisted of 4 systematic reviews and a meta-analysis consisting of 11 RCTs and 10 observational studies that aimed to compare IV metoprolol versus diltiazem in patients with AF with RVR. In terms of effectiveness outcomes, IV diltiazem therapy was associated with a notably more substantial decrease in ventricular rate when compared to IV metoprolol. Nevertheless, the most pronounced reduction occurred at 10 min rather than at 30 or 60 min. Additionally, our investigation indicated that the administration of IV diltiazem was associated with a notably increased likelihood of achieving successful rate control in patients with AF with RVR compared to IV metoprolol. On the contrary, Bosch et al.^29^ conducted a study in the intensive care unit (ICU) to compare the efficacy of IV calcium channel blockers and β-blockers in managing AF accompanied by RVR in the context of sepsis. The results indicated that IV calcium channel blockers were less effective than IV β-blockers in acute rate control within 1 h. However, this disparity did not reach statistical significance. Interestingly, the investigation by Moskowitz et al.,^30^ which was encompassed in our comprehensive review, similarly identified a statistically significant inferiority in rate control with calcium channel blockers compared to β-blockers (odds ratio, 0.64; 95% CI, 0.45–0.92). Consequently, the findings from these two studies conducted in the ICU setting suggest that, within the ICU population, β-blockers might be a more favorable choice than calcium channel blockers for acute rate control. However, it is essential to note that there is a lack of randomized trial evidence to substantiate this observation, and our study demonstrated a distinct preference for diltiazem as the treatment option over metoprolol in our analysis.

Likewise, a research investigation by Lan et al.^20^ documented that IV diltiazem demonstrated superior efficacy in decreasing the ventricular rate; this suggests that diltiazem leads to a more conspicuous ventricular rate deceleration and shortened onset time. Regarding diastolic blood pressure and adverse events, IV diltiazem and metoprolol did not differ significantly. However, it was noted that IV metoprolol may lead to a reduction in systolic blood pressure. As a result, IV diltiazem effectively regulates the ventricular rate of patients presenting to an ED with AF with RVR, providing more significant benefits and fewer adverse events for health care professionals.

Our umbrella review improves validity by integrating all systematic reviews and meta-analyses comparing IV diltiazem versus metoprolol, including a diverse set of RCTs and observational data. Our study compares acute rate control, ventricular rate, and numerous outcomes (hypotension, bradycardia, adverse events, and systolic and diastolic blood pressures) with different dosages, arrhythmia types, and treatment intervals, thereby enhancing its credibility. Our study adds to the literature by carefully detecting and assessing inconsistencies across previous studies, providing a critical appraisal of evidence. Furthermore, we investigate the time-dependent efficacy of IV diltiazem and metoprolol, with a particular emphasis on the critical 10-min mark, offering practical insights for clinical decision-making. The focus on the increased risk of hypotension with IV diltiazem adds a safety factor to the current research, increasing its granularity. Beyond summarizing research, our work proposes developing standardized decision-making guidelines for physicians based on synthesis evidence, providing a proactive and relevant addition to the present debate.

It is also crucial to acknowledge our limitations. The variation in results between systematic reviews can be due to variances in methodology, inclusion criteria, and outcome measures. Furthermore, inherent biases may occur as a result of the comprehensive scope of these studies and their dependence on available data. Our review does not include any newly created data because it is based purely on previously collected data. Umbrella reviews, which include systematic reviews and meta-analyses, need help proving causal correlations, particularly with observational research that may consist of confounding variables. Despite a thorough search, there are few studies directly comparing the two medicines in the context of a “wait and see” strategy, particularly in patients undergoing cardioversion. Finding relevant research for this comparison was challenging. Furthermore, none of the included trials specifically commented on the rate of sinus rhythm restoration, owing to their primary focus on rate control, despite the fact that both metoprolol and diltiazem are known rate-controlling drugs.

## Conclusion

In conclusion, IV diltiazem therapy demonstrated its superiority over metoprolol in managing AF with RVR, leading to greater efficacy in rate control and a substantial reduction in ventricular rate. Further research, particularly clinical trials, should be undertaken to compare the overall effectiveness and safety profile of calcium channel blockers and β-blockers in regulating HR, considering the adverse events associated with the former.

## Figures and Tables

**Table 1:** Characteristics of Included Meta-analyses and Systematic Reviews

Study (year)	Lan et al. (2022)^20^	Bolton et al. (2024)^21^	Sharda and Bhatia (2022)^22^	Jafri et al. (2021)^23^
Study type	Meta-analysis	Meta-analysis	Systematic review and meta-analysis	Systematic review and meta-analysis
Total no. of patients	1214	2158	1732	140
Patients in the diltiazem group, n (%)	643 (52.9)	985 (45.6)	773 (44.6)	68 (48.5)
Patients in the metoprolol group, n (%)	571 (47)	1173 (54.3)	959 (55.3)	72 (51.4)
Dose of diltiazem	0.25 mg/kg (maximum, 25 mg)	0.25 mg/kg (maximum, 25 mg)	0.25 mg/kg IV (maximum, 30 mg) followed by 0.35 mg/kg (maximum, 30 mg) if required after 15 min	0.25 mg/kg (maximum, 25 mg)
Dose of metoprolol	0.15 mg/kg (maximum, 10 mg)	0.15 mg/kg (maximum, 10 mg)	0.15 mg/kg IV (maximum, 10 mg) followed by 0.25 mg/kg (maximum, 10 mg) if required after 15 min	0.15 mg/kg (maximum, 10 mg)
Arrhythmia studied	AF	AF	AF/atrial flutter	AF
Primary outcomes	Achievement of rate-control target at 5, 10, 30, and 60 min; average onset time; and decrease in ventricular rate	Achievement of rate-control target at 5, 10, 30, and 60 min	Achievement of rate-control target at 5, 10, 30, and 60 min	Achievement of rate-control target at 10 min
Secondary outcomes	Systolic blood pressure, diastolic blood pressure, adverse events	Hypotension	Hypotension and bradycardia	Mean systolic blood pressure at 15 min

*Abbreviation:* AF, atrial fibrillation.

**Supplementary Table S1:** Search Strategy

Database	Search Strategy	Number of Articles Found
PubMed	(“atrial fibrillation”[MeSH Terms] OR (“atrial”[All Fields] AND “fibrillation”[All Fields]) OR “atrial fibrillation”[All Fields] OR (“atrial fibrillation”[MeSH Terms] OR (“atrial”[All Fields] AND “fibrillation”[All Fields]) OR “atrial fibrillation”[All Fields] OR “afib”[All Fields]) OR ((“atrialisation”[All Fields] OR “atrialization”[All Fields] OR “atrialized”[All Fields] OR “atrially”[All Fields] OR “heart atria”[MeSH Terms] OR (“heart”[All Fields] AND “atria”[All Fields]) OR “heart atria”[All Fields] OR “atrial”[All Fields]) AND (“arrhythmia s”[All Fields] OR “arrhythmias, cardiac”[MeSH Terms] OR (“arrhythmias”[All Fields] AND “cardiac”[All Fields]) OR “cardiac arrhythmias”[All Fields] OR “arrhythmia”[All Fields] OR “arrhythmias”[All Fields]))) AND (((“rapid”[All Fields] OR “rapidities”[All Fields] OR “rapidity”[All Fields] OR “rapidness”[All Fields]) AND (“heart ventricles”[MeSH Terms] OR (“heart”[All Fields] AND “ventricles”[All Fields]) OR “heart ventricles”[All Fields] OR “ventricular”[All Fields] OR “ventricularization”[All Fields] OR “ventricularized”[All Fields]) AND (“j rehabil assist technol eng”[Journal] OR “rate”[All Fields])) OR (“tachycardia”[MeSH Terms] OR “tachycardia”[All Fields] OR “tachycardias”[All Fields] OR “tachycardia s”[All Fields])) AND (((“intravenous”[All Fields] OR “intraveneously”[All Fields] OR “intravenous”[All Fields] OR “intravenously”[All Fields]) AND (“diltiazem”[MeSH Terms] OR “diltiazem”[All Fields])) OR (“calcium channel blockers”[Pharmacological Action] OR “calcium channel blockers”[MeSH Terms] OR (“calcium”[All Fields] AND “channel”[All Fields] AND “blockers”[All Fields]) OR “calcium channel blockers”[All Fields] OR (“calcium”[All Fields] AND “channel”[All Fields] AND “blocker”[All Fields]) OR “calcium channel blocker”[All Fields]) OR (“diltiazem”[MeSH Terms] OR “diltiazem”[All Fields] OR (“diltiazem”[All Fields] AND “hydrochloride”[All Fields]) OR “diltiazem hydrochloride”[All Fields])) AND (“metoprolol”[MeSH Terms] OR “metoprolol”[All Fields] OR (“adrenergic beta antagonists”[Pharmacological Action] OR “adrenergic beta antagonists”[MeSH Terms] OR (“adrenergic”[All Fields] AND “beta antagonists”[All Fields]) OR “adrenergic beta antagonists”[All Fields] OR (“beta”[All Fields] AND “blocker”[All Fields]) OR “beta blocker”[All Fields]) OR (“metoprolol”[MeSH Terms] OR “metoprolol”[All Fields] OR “toprol”[All Fields]))	10
Cochrane Library	(Atrial fibrillation OR AFib OR atrial arrhythmia) AND (rapid ventricular rate OR tachycardia) AND (Intravenous diltiazem OR calcium channel blocker OR Diltiazem hydrochloride) AND (Metoprolol OR beta-blocker OR Toprol)	7
Scopus	(Atrial fibrillation OR AFib OR atrial arrhythmia) AND (rapid ventricular rate OR tachycardia) AND (Intravenous diltiazem OR calcium channel blocker OR Diltiazem hydrochloride) AND (Metoprolol OR beta-blocker OR Toprol)	3

*Abbreviation:* MeSH, Medical Subject Heading.

**Supplementary Table S2:** Assessing the Methodological Quality of Systematic Reviews—AMSTAR 2

References	AMSTAR 2 Items^a^																Overall Rating^b^
	1	2	3	4	5	6	7	8	9	10	11	12	13	14	15	16	
Lan et al. (2022)^20^	No	Yes	Yes	PY	Yes	Yes	No	Yes	Yes	No	Yes	No	No	Yes	No	Yes	Moderate
Bolton et al. (2024)^21^	Yes	Yes	Yes	Yes	Yes	Yes	Yes	Yes	Yes	Yes	Yes	No	Yes	Yes	No	Yes	Moderate
Sharda and Bhatia (2022)^22^	Yes	Yes	No	Yes	Yes	Yes	Yes	Yes	Yes	No	Yes	No	Yes	Yes	Yes	No	Moderate
Jafri et al. (2021)^23^	Yes	Yes	No	Yes	Yes	Yes	Yes	Yes	Yes	Yes	Yes	Yes	Yes	No	Yes	No	Moderate
Total amount of “yes” responses	3	4	2	3	4	4	3	4	4	2	4	2	3	3	2	2	

*Abbreviations:* PECO, Population, Exposure, Comparison, Outcome; PICO, Population, Intervention, Comparator, and Outcome; PY, partial yes; RoB, risk of bias.

^a^AMSTAR items:
Did the research questions and inclusion criteria for the review include the components of PICO/PECO? Did the report of the review contain an explicit statement that the review methods were established prior to the conduct of the review and did the report justify any significant deviations from the protocol? Did the review authors explain their selection of the study designs for inclusion in the review? Did the review authors use a comprehensive literature search strategy? Did the review authors perform study selection in duplicate? Did the review authors perform data extraction in duplicate? Did the review authors provide a list of excluded studies and justify the exclusions? Did the review authors describe the included studies in adequate detail? Did the review authors use a satisfactory technique for assessing the RoB in individual studies that were included in the review? Did the review authors report on the sources of funding for the studies included in the review? If a meta-analysis was performed did the review authors use appropriate methods for statistical combination of results? If a meta-analysis was performed, did the review authors assess the potential impact of RoB in individual studies on the results of the meta-analysis or other evidence synthesis? Did the review authors account for RoB in individual studies when interpreting/discussing the results of the review? Did the review authors provide a satisfactory explanation for, and discussion of, any heterogeneity observed in the results of the review? If they performed a quantitative synthesis, did the review authors carry out an adequate investigation of publication bias (small-study bias) and discuss its likely impact on the results of the review? Did the review authors report any potential sources of conflict of interest, including any funding they received for conducting the review?

^b^Rating overall confidence in the results of the review:
High: No or one non-critical weakness; the systematic review provides an accurate and comprehensive summary of the results of the available studies that address the question of interest. Moderate: More than one non-critical weakness^c^; the systematic review has more than one weakness but no critical flaws. It may provide an accurate summary of the results of the available studies that were included in the review. Low: One critical flaw with or without non-critical weaknesses; the review has a critical flaw and may not provide an accurate and comprehensive summary of the available studies that address the question of interest. Critically low: More than one critical flaw with or without non-critical weaknesses; the review has more than one critical flaw and should not be relied on to provide an accurate and comprehensive summary of the available studies.

^c^Multiple non-critical weaknesses may diminish confidence in the review, and it may be appropriate to move the overall appraisal down from moderate to low confidence.

Adapted from Shea et al. (2017).^13^

**Supplementary Table S3A:** Grade Assessment of the Meta-analyses and Systematic Reviews Included in Lan et al. (2022)^20^ According to the Comparison of Intravenous Diltiazem and Metoprolol for Atrial Fibrillation

Certainty Assessment							No. of Patients		Effect		Certainty	Importance
No. of Studies	Study Design	Risk of Bias	Inconsistency	Indirectness	Imprecision	Other Considerations	Diltiazem	Metoprolol	Relative (95% CI)	Absolute (95% CI)		
Rate control at 5 min												
17	9 randomized and 8 non-randomized studies	Not serious	Not serious	Not serious	Not serious	None	127/643 (19.8%)	115/571 (20.1%)	RR, 0.98 (0.78–1.23)	4 fewer per 1000 (from 44 fewer to 46 more)	⨁⨁⨁⨁ High	CRITICAL
Rate control at 10 min												
17	9 randomized and 8 non-randomized studies	Not serious	Not serious	Not serious	Not serious	None	127/643 (19.8%)	120/571 (21.0%)	RR, 0.94 (0.75–1.17)	13 fewer per 1000 (from 53 fewer to 36 more)	⨁⨁⨁⨁ High	CRITICAL
Rate control at 30 min												
17	9 randomized and 8 non-randomized studies	Not serious	Not serious	Not serious	Not serious	None	216/643 (33.6%)	178/571 (31.2%)	RR, 1.08 (0.92–1.27)	25 more per 1000 (from 25 fewer to 84 more)	⨁⨁⨁⨁ High	IMPORTANT
Rate control at 60 min												
17	9 randomized and 8 non-randomized studies	Not serious	Not serious	Not serious	Not serious	None	229/643 (35.6%)	183/571 (32.0%)	RR, 1.11 (0.95–1.30)	35 more per 1000 (from 16 fewer to 96 more)	⨁⨁⨁⨁ High	CRITICAL

*Abbreviations:* AF, atrial fibrillation; CI, confidence interval; RR, risk ratio.

**Supplementary Table S3B:** Grade Assessment of the Meta-analyses and Systematic Reviews Included in Bolton et al. (2024)^21^ According to the Comparison of Intravenous Diltiazem and Metoprolol for Atrial Fibrillation

Certainty Assessment							No. of Patients		Effect		Certainty	Importance
No. of Studies	Study Design	Risk of Bias	Inconsistency	Indirectness	Imprecision	Other Considerations	Diltiazem	Metoprolol	Relative (95% CI)	Absolute (95% CI)		
Rate control at 5 min												
16	7 randomized and 9 non-randomized studies	Serious	Not serious	Not serious	Not serious	None	985	1173	RR, 2.08 (1.04–4.18)	0 fewer per 1000 (from 0 fewer to 0 fewer)	⨁⨁⨁◯ Moderate	CRITICAL
Rate control at 10 min												
16	7 randomized and 9 non-randomized studies	Serious	Not serious	Not serious	Not serious	None	985	1173	RR, 1.55 (0.84–2.87)	0 fewer per 1000 (from 0 fewer to 0 fewer)	⨁⨁⨁◯ Moderate	CRITICAL
Rate control at 30 min												
16	7 randomized and 9 non-randomized studies	Serious	Not serious	Not serious	Not serious	None	985	1173	RR, 3.77 (1.70–8.35)	0 fewer per 1000 (from 0 fewer to 0 fewer)	⨁⨁⨁◯ Moderate	CRITICAL
Rate control at 60 min												
16	7 randomized and 9 non-randomized studies	Serious	Not serious	Not serious	Not serious	None	985	1173	RR, 5.78 (2.60–12.80)	0 fewer per 1000 (from 0 fewer to 0 fewer)	⨁⨁⨁◯ Moderate	CRITICAL
Hypotension												
16	7 randomized and 9 non-randomized studies	Serious	Not serious	Not serious	Not serious	None	985	1173	RR, 1.12 (0.51–2.45)	0 fewer per 1000 (from 0 fewer to 0 fewer)	⨁⨁⨁◯ Moderate	IMPORTANT

*Abbreviations:* AF, atrial fibrillation; CI, confidence interval; RR, risk ratio.

**Supplementary Table S3C:** Grade Assessment of the Meta-analyses and Systematic Reviews Included in Sharda and Bhatia (2022)^22^ According to the Comparison of Intravenous Diltiazem and Metoprolol for Atrial Fibrillation

Certainty Assessment							No. of Patients		Effect		Certainty	Importance
No. of Studies	Study Design	Risk of Bias	Inconsistency	Indirectness	Imprecision	Other Considerations	Diltiazem	Metoprolol	Relative (95% CI)	Absolute (95% CI)		
Rate control at 60 min												
14	11 randomized and 3 nonrandomized studies	Serious	Not serious	serious	Not serious	All plausible residual confounding would reduce the demonstrated effect	481/773 (62.2%)	478/959 (49.8%)	RR, 1.25 (1.15–1.36)	125 more per 1000 (from 75 more to 179 more)	⨁⨁⨁◯ Moderate	CRITICAL
Hypotension												
14	11 randomized and 3 nonrandomized studies	Serious	Not serious	Serious	Not serious	All plausible residual confounding would reduce the demonstrated effect	131/773 (16.9%)	111/959 (11.6%)	RR, 1.46 (1.16–1.85)	53 more per 1000 (from 19 more to 98 more)	⨁⨁⨁◯ Moderate	IMPORTANT

*Abbreviations:* AF, atrial fibrillation; CI, confidence interval; RR, risk ratio.

**Supplementary Table S3D:** Grade Assessment of the Meta-analyses and Systematic Reviews Included in Jafri et al. (2021)^23^ According to the Comparison of Intravenous Diltiazem and Metoprolol for Atrial Fibrillation

Certainty Assessment							No. of Patients		Effect		Certainty	Importance
No. of Studies	Study Design	Risk of Bias	Inconsistency	Indirectness	Imprecision	Other Considerations	Diltiazem	Metoprolol	Relative (95% CI)	Absolute (95% CI)		
Rate control at 10 min												
3	Randomized trials	Not serious	Not serious	Not serious	Not serious	None	68	72	RR, 2.14 (1.01–4.54)	0 fewer per 1000 (from 0 fewer to 0 fewer)	⨁⨁⨁⨁ High	IMPORTANT

*Abbreviations:* AF, atrial fibrillation; CI, confidence interval; RR, risk ratio.

**Supplementary Table S4:** The Newcastle–Ottawa Scale

Study	Selection				Comparability	Outcomes			Total
	Representativeness of the Exposed Cohort	Selection of the Non-exposed Cohort	Ascertainment of Exposure	Demonstration That Outcome of Interest Was Not Present at Start of Study	Comparability of Cohorts on the Basis of the Design or Analysis	Assessment of Outcome	Was Follow-up Long Enough for Outcomes to Occur	Adequacy of Follow-up of Cohorts	
Compagner (2022)	*	*	*	*	**	*	*	*	*********
Hasbrouck (2022)	*	*	*	*	*	*	*	*	********
Hines (2016)	*	*	*	*	*	*	*	*	********
Hirschy (2018)	*	*	*	-	*	*	*	*	******
McGrath (2020)	*	*	*	*	**	*	*	*	*********
Medeiros (2021)	*	*	*	*	*	*	*	*	********
Moskowitz et al. (2017)	*	*	*	*	*	*	*	*	********
Nuñez Cruz (2020)	*	*	*	-	*	*	*	*	******
Xiao (2022)	*	*	*	*	**	*	*	*	*********
Zhang (2009)	*	*	*	*	*	*	*	*	********
Diao (2009)	*	*	*	-	*	*	*	*	******
Fan Shuxiong (2012)	*	*	*	*	**	*	*	*	*********
Nicholson (2020)	*	*	*	*	*	*	*	*	********
Hargrove et al. (2021)	*	*	*	-	*	*	*	*	******
Demir (2021)	*	*	*	*	**	*	*	*	*********
Feeney (2018)	*	*	*	*	*	*	*	*	********
Katchi (2014)	*	*	*	-	*	*	*	*	******
Memis (2018)	*	*	*	*	**	*	*	*	*********
Personett (2014)	*	*	*	*	*	*	*	*	********

This table thoroughly assesses the quality of observational studies included in the meta-analyses that were pooled for this meta-analysis using the Newcastle–Ottawa scale. The evaluation delineates a spectrum of study quality, ranging from fair to good. In the Newcastle–Ottawa scale, an asterisk (*) signifies a point awarded for meeting specific criteria, with good quality defined as 3 or 4 stars in the selection domain AND 1 or 2 star(s) in the comparability domain AND 2 or 3 stars in the outcome/exposure domain. Fair quality is characterized by 2 stars in the selection domain, 1 OR 2 star(s) in the comparability domain, AND 2 or 3 stars in the outcome/exposure domain. Poor quality is indicated by 0 or 1 star(s) in the selection domain, 0 stars in the comparability domain, OR 0 or 1 star(s) in the outcome/exposure domain.
